# Hippocampal Lactate-Infusion Enhances Spatial Memory Correlated with Monocarboxylate Transporter 2 and Lactylation

**DOI:** 10.3390/brainsci14040327

**Published:** 2024-03-28

**Authors:** Yuhan Wu, Hui Hu, Weiwei Liu, Yun Zhao, Fang Xie, Zhaowei Sun, Ling Zhang, Huafeng Dong, Xue Wang, Lingjia Qian

**Affiliations:** 1 Beijing Institute of Basic Medical Sciences, Academy of Military Medical Sciences, Beijing 100039, China; wyhan0204@163.com (Y.W.); will2mars@163.com (H.H.); lww168502@163.com (W.L.); zhaoyun@bmi.ac.cn (Y.Z.); vancoxie@sina.com (F.X.); sunzhw0820@163.com (Z.S.); zl18306826208@163.com (L.Z.); feng13191908265@163.com (H.D.); 2 College of Public Health, North China University of Science and Technology, Tangshan 063210, China; 3 Institute of Military Cognition and Brain Sciences, Beijing 100850, China

**Keywords:** lactate, spatial memory, MCT2, lactylation, HT22

## Abstract

Lactate has emerged as a key player in regulating neural functions and cognitive processes. Beyond its function as an energy substrate and signal molecule, recent research has revealed lactate to serve as an epigenetic regulator in the brain. However, the molecular mechanisms by which lactate regulates spatial memory and its role in the prevention of cognitive disorders remain unclear. Herein, we injected L-lactate (10 μmol/kg/d for 6 d) into the mouse’s hippocampus, followed by the Morris water maze (MWM) test and molecular analyses. Improved spatial memory performances were observed in mice injected with lactate. Besides, lactate upregulated the expression of synaptic proteins post-synaptic density 95 (PSD95), synaptophysin (SYP), and growth associated protein 43 (GAP43) in hippocampal tissues and HT22 cells, suggesting a potential role in synaptic transmission and memory formation. The facilitative role of monocarboxylate transporter 2 (MCT2), a neuron-specific lactate transporter, in this process was confirmed, as MCT2 antagonists attenuated the lactate-induced upregulation of synaptic proteins. Moreover, lactate induced protein lactylation, a post-translational modification, which could be suppressed by MCT2 inhibition. RNA sequencing of lactated-injected hippocampal tissues revealed a comprehensive gene expression profile influenced by lactate, with significant changes in genes associated with transcriptional progress. These data demonstrate that hippocampal lactate injection enhances spatial memory in mice, potentially through the upregulation of synaptic proteins and induction of protein lactylation, with MCT2 playing a crucial role in these processes. Our findings shed light on the multi-faceted role of lactate in neural function and memory regulation, opening new avenues for therapeutic interventions targeting cognitive disorders.

## 1. Introduction

The capacity to learn and retain spatial locations and link them with other stimuli is a fundamental adaptive behavior crucial for survival, and relies on complex neural processes within the brain. Among the myriad brain regions implicated in these processes, the hippocampus stands out as a pivotal structure closely associated with the encoding, consolidation, and retrieval of spatial information [1]. It has been widely suggested that spatial memories are stored as changes in the synaptic connections between neurons [2,3]. This intricate process involves the participation of various molecular players, including synaptic proteins, such as post-synaptic density 95 (PSD95), Synaptophysin (SYP), and growth-associated protein 43 (GAP43). These proteins are integral to synaptic function and are critical for the formation and maintenance of synaptic plasticity [4,5,6]. The impairment of hippocampal synaptic function and reduced expression of synaptic proteins have been found in cognitive disorders, including neuro-degenerative diseases and stress-related cognitive impairment [7]. Therefore, the identification of novel targets and strategies to enhance hippocampal function holds significant importance in improving spatial memory ability and intervening in cognitive diseases.

Lactate, as a small molecule produced during cellular metabolism, has come a long way from cellular waste to cellular fuel and inter-cellular messenger. Recent advances in research have shown that lactate exerts many positive biological functions in the brain [8]. Lactate is involved in the regulation of the physiological activity of the brain and is indispensable for the development and function of the central nervous system [9,10,11]. Notably, brain lactate has been implicated as a vital mediator of cognitive function. Studies have reported a substantial elevation in hippocampal lactate levels following learning and an essential role of lactate released by astrocytes in the formation and maintenance of long-term memory formation [12]. A significant reduction in levels of parenchymal lactate and monocarboxylate transporter 2 (MCT2) expression in the brain was found in Alzheimer’s disease mice [11]. Furthermore, ameliorating brain lactate levels or lactate metabolism has been shown to enhance synaptic plasticity, neurotrophic factor levels and, ultimately, promote brain function [13], indicating a potential role of lactate in the prevention of cognitive diseases.

Lactate serves not only metabolic functions but also regulates various cellular processes in multiple ways [8]. In recent years, lactylation modification has been found to be an important way for lactate to perform biological functions [14,15]. Lactate can be covalently modified to protein lysine residues as a substrate and is involved in epigenetic regulation [16,17]. It has been found that protein lactylation is involved in glycolysis-related cell functions [18], macrophage polarization [19], tumor proliferation regulation [20], nervous system regulation [21] and other important life activities. Furthermore, neural excitation can induce lactylation modification of proteins in brain cells [22]. However, our understanding of the molecular mechanisms through which lactate exerts its effects in the brain is still at an early stage, necessitating further exploration.

In the present study, we investigated the effect of lactate on spatial memory and its molecular mechanisms in mice. Improved spatial memory performance and increased expression of synaptic proteins PSD95, SYP, and GAP43 were observed after hippocampal lactate administration. Additionally, lactate transporter MCT2 was demonstrated to be involved in regulating the function of lactate in hippocampal neurons. Furthermore, we found that lactate induced protein lactylation and elucidated the gene expression profile and key biological progresses influenced by lactate in the hippocampus. These findings may provide a new clue for the treatment of cognitive impairment.

## 2. Materials and Methods

### 2.1. Animals

C57BL6 mice were used for biochemical and behavioral studies, and were purchased from SBF Biotechnology Co., Ltd. (Beijing, China). All mice were adult males. Animals were kept with normal circadian rhythms and diet and housed in groups of three or four under standard conditions. All mice were handled for 5 min per day for 6 days prior to any procedure.

### 2.2. L-Lactate Injection

**Figure 1:**
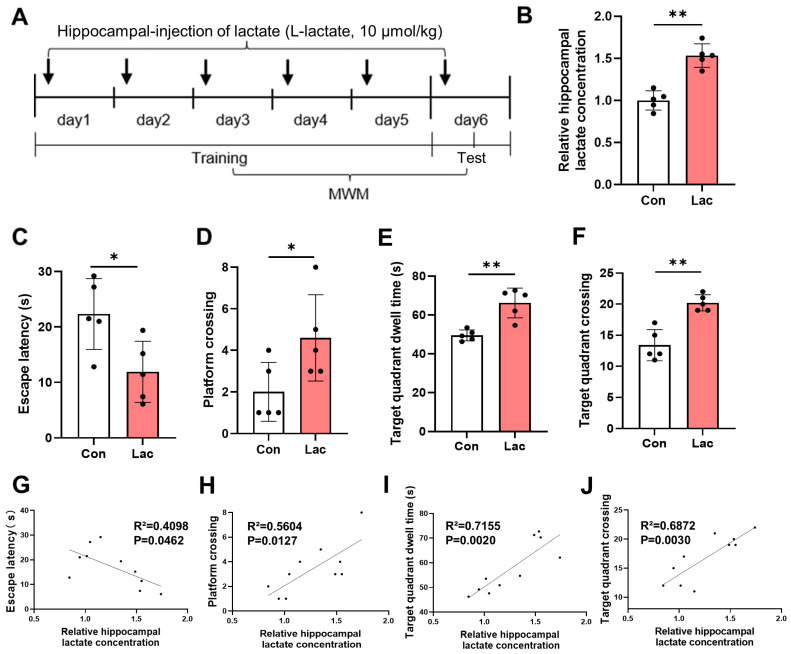
Lactate improves spatial memory ability of mice. (**A**) Schematic of lactate infusion into the hippocampus and experimental time-line. Mice infused with PBS were used as a control. (**B**) Relative concentrations of lactate in the brain tissues of control and lactate injected mice (*n* = 5, Student’s *t*-test, ** *p* < 0.01). (**C**–**F**) Latency to enter the platform, platform crossings, target quadrant dwell time, and target quadrant crossings of mice in the MWM (*n* = 5, Student’s *t*-test, * *p* < 0.05; ** *p* < 0.01). (**G**–**J**) Correlation analysis between the results of MWM and the concentration of lactate in the hippocampus of mice (*n* = 5, Simple linear regression).

Phosphate-buffered saline (PBS, pH 7.4, concentration of 0.2 mol/L) was used to dissolve sodium L-lactate (Aladdin, Shanghai, China) as previously reported [23]. The cannula (RWD Life Science, Shenzhen, China) was fixed 1.5 mm below the skull, located 1.9 mm behind the fontanelle and 1.9 mm from the mid-line. The infusion needles extended 0.5 mm beyond the cannula reaching the hippocampal position. All injections were performed at 10 μmol/kg bodyweight per hippocampal side and the controls were injected with the same volume of PBS. At 1 h before Morris water maze (MWM) training, the solution was injected into the hippocampus of mice (0.25 μL/min) with an infusion pump (Figure 1A). After the injection, the needle was kept in place for one minute to ensure that the solution was fully dispersed.

### 2.3. Lactate Measurement

The hippocampal tissues of mice or cells were collected and homogenized in an ice bath. According to the instructions, lactate content was detected using the CheKine™ Lactate Assay Kit (Abbkine, Gaithersburg, MD, USA). In this kit, lactate is oxidized by lactate dehydrogenase to produce a product that interacts with the tetrazolium salt WST-8 dye, forming a colored product proportional to the lactate concentration in the sample with a maximum absorption peak at 450 nm. The data were analyzed through the standard curve and the calculation formula.

### 2.4. Morris Water Maze

A black tank (diameter, 160 cm; depth, 75 cm) containing water (22 ± 2 °C) was used in this experiment. Titanium dioxide was added to water to improve contrast. Spatial reference markers in the form of stickers of various shapes and colours were affixed around the tank. Mice underwent 5 half-day training sessions, once a day for 3 min each. Before each training session, lactate or PBS was injected into the hippocampus of mice through the cannula. Mice were put in one quadrant to start training and to find the submerged platform (1 cm beneath the water), were guided to the platform once they had not found the platform within the time limit (3 min) and kept on the platform for 10 s to create a memory. Finally, we removed the platform and placed mice on the opposite quadrant, swimming in the water for 3 min. The time when the mice found the original platform location was recorded as “Escape latency”. The total number of platform crossings in the limited time was recorded as “Platform crossing”. The number of crossings to the quadrant where the platform was located and the time spent in that quadrant were recorded as “Target quarter crossing” and “Target quarter dwell time”. The above data were recorded and further analyzed.

### 2.5. Cell Culture and Intervention

The neuronal cell line from the mouse hippocampal, HT22, was obtained from BeNa Culture Collection (Beijing, China). Cells were cultured using complete medium (5% CO_2_, 37 °C). Complete medium was configured by 10% fetal bovine serum (FBS) (TIANHANG, Hangzhou, China), 90% DMEM (Sigma, St. Louis, MO, USA) and penicillin and streptomycin (100 U/mL, Solarbio, Beijing, China). Cells were seeded at 1.5 × 10^5^/mL in plates and allowed to grow when the bottom of the dish reached 90% confluence, treated with added lactate (4 mmol/L), AR-C155858 (10 and 20 μmol/L) (AR-C, MCE, Monmouth Junction, NJ, USA) or α-cyano-4-hydroxycinnamic acid (100 and 200 μmol/L) (α-CHCA, Sigma-Aldrich, St. Louis, MO, USA) for 24 h refer as previous reported [23].

### 2.6. RNA Reverse-Transcription and Real-Time Quantitative Polymerase Chain Reaction (qPCR) Analysis

Trizol reagent (Sigma, St. Louis, MO, USA) was selected to extract cellular RNA. An appropriate amount of Trizol was added according to cell volume and tissue volume, 200 μL trichloromethane, 600 μL isopropyl alcohol and 75% ethanol (DEPC water configuration) were added in sequence per 1 mL Ttizol, incubated and centrifuged, and RNA was extracted. PrimeScriptTMRT Master Mix (Takara, Osaka, Japan) was used to transcribed total RNA (2 μg) into cDNA. qPCR was run using TB Green Premix Ex Taq II (Takara, Osaka, Japan). Briefly, the qPCR reaction system volume was 10.0 µL, with 0.2 µL each of forward and reverse primers, 1.0 µL of cDNA sample, and 5.0 µL of TB Green II. The thermal cycling parameters included initial denaturation for 95 °C (2 min), 40 cycles of 95 °C (10 s), 60 °C (10 s) and 72 °C (15 s) For consistency, we used β-actin gene as an endogenous control for normalization of total RNA levels in each sample. The indicated gene relative levels were calculated by the 2^−ΔΔCt^ method as described previously [24]. The primer sequences are listed as Supplementary Table S1.

### 2.7. Western Blot Analysis

Cell and tissue protein lysed in RIPA (Thermo, Waltham, MA, USA) was supplemented with a protease inhibitors cocktail (Biosharp, Shanghai, China), while bromophenol blue and elevated temperature caused protein denaturation. Protein was separated by poly acrylamide TGX gel (Vazyme, Nanjing, China) by sodium lauryl sulfate polyacrylamide gel electrophoresis, and the protein transferred to a polyvinylidene fluoride (PVDF) membrane. The membrane was exposed to primary antibodies in a Tris Buffered Saline (TBS) with Tween 20 at 4 °C overnight after blocking with 10% milk and incubated in the secondary antibody for 2 h. The Image Quant™ LAS 4000 imaging system (GE, Boston, MA, USA) and enhanced chemiluminescence western blot detection reagent (Vazyme, Nanjing, China) were utilized. The antibodies are listed in Supplementary Table S2.

### 2.8. RNA-Seq and Differentially Expressed Gene (DEG) Analysis

Tissue’s total RNA was extracted using Trizol reagent (Plant RNA Purification Reagent for plant tissue). Genomic DNA was removed using DNase I. Shanghai Majorbio Bio-pharm Biotechnology Co., Ltd. (Shanghai, China) performed RNA purification, reverse transcription, library construction, and sequencing. Total RNA (1 µg) was used to prepare the transcriptome library by the TruSeq TM RNA sample preparation Kit from Illumina (San Diego, CA, USA). The gene expression level was calculated using transcripts per million reads method. In addition, we quantify gene abundances by the RSEM website. To identify enriched terms and pathways for the DEGs, functional-enrichment analysis was performed. These analyses were based on Goatools, KOBAS, and DAVID databases.

### 2.9. Data Analysis

The GraphPad Prism 8.0 was used and data was presented as mean ± SEM. Normality of continuous variables was evaluated. To assess the significance of differences between two independent groups, a two-tailed unpaired Student’s *t*-test was used. One-way ANOVA was used for the comparisons of more than four groups, followed by Tukey’s post-hoc analysis. Pearson’s correlation analysis was performed to determine the correlation between two variables. *p* < 0.05 was considered to be significant.

## 3. Results

### 3.1. Hippocampal Injection of Lactate Improves the Spatial Memory of Mice

To assess the impact of lactate levels in the hippocampus on spatial memory ability, lactate was injected bilaterally into the hippocampus of mice, followed by the MWM test (Figure 1A). The elevated hippocampal lactate level after injection was confirmed (Figure 1B). Besides, the lactate-injected mice exhibited reduced swimming time to reach the platform (Figure 1C), increased number of platform crossings (Figure 1D), longer time spent in the target quadrant (Figure 1E), and higher number of target quadrant crossings (Figure 1F) compared to the control group in the MWM test, indicating increased spatial memory ability. Correlation analysis was conducted to determine the relationship between hippocampal lactate levels and cognitive behaviors. The hippocampal lactate levels showed a negative correlation with the escape latency (Figure 1G) and a positive correlation with the number of platform crossings (Figure 1H), time spent in the target quadrant (Figure 1I), and number of target quadrant crossings (Figure 1J) in the MWM test. Overall, the data indicate that elevated level of lactate in the hippocampal promotes the spatial memory ability of mice.

### 3.2. Lactate Increases the Expression of Synaptic Proteins

**Figure 2:**
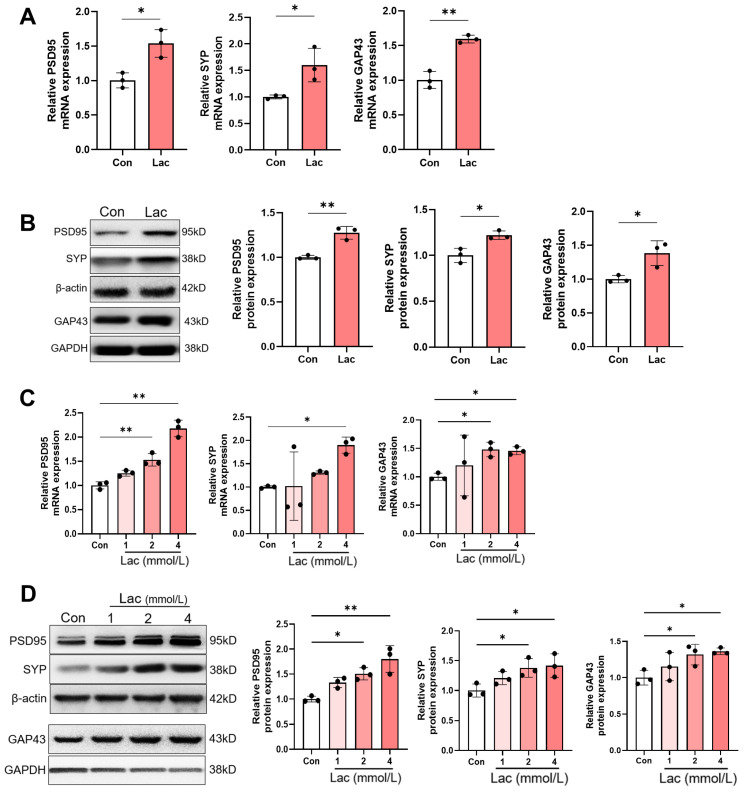
Lactate participates in regulating the expression of synaptic function-related proteins. (**A**) qRT–PCR assays monitoring the expression of PSD95, SYP, and GAP43 in hippocampal samples from control and lactate injected mice (*n* = 3, Student’s *t*-test, * *p* < 0.05; ** *p* < 0.01). (**B**) Western blot of the expression of PSD95, SYP, and GAP43 in hippocampal samples from control and lactate injected mice (*n* = 3, Student’s *t*-test, * *p* < 0.05; ** *p* < 0.01). (**C**) qRT–PCR assays monitoring the expression of PSD95, SYP, and GAP43 in lactate –treated (1 mM, 2 mM, and 4 mM) HT22 cells and control cells (*n* = 3, Ordinary one-way ANOVA, * *p* < 0.05; ** *p* < 0.01). (**D**) Western blot of the expression of PSD95, SYP, and GAP43 in lactate-treated (1 mM, 2 mM, and 4 mM) HT22 cells and control cells (*n* = 3, Ordinary one-way ANOVA, * *p* < 0.05; ** *p* < 0.01).

The expression of synaptic proteins is essential for synaptic transmission, which plays a crucial role in memory formation and retention [7,25]. Therefore, we explored the impact of lactate on the expression of PSD95, SYP and GAP43. As shown in Figure 2A, the mRNA levels of PSD95, SYP, and GAP43 were upregulated in the hippocampal tissues following injection of lactate versus the controls. Western blot results also revealed an increase in the protein levels of PSD95, SYP, and GAP43, which further confirmed a positive role of lactate on the expression of synaptic proteins (Figure 2B). We next used the hippocampal HT22 cell line for validation of the biological function of lactate at the cellular level. After intervention with different concentrations of lactate (1, 2, and 4 mmol/L), the expression of PSD95, SYP, and GAP43 in HT22 cells were detected. The results of qRT–PCR showed that a lactate concentration of 2 mmol/L induced the expression of PSD95 and GAP43, while a concentration of 4 mmol/L induced the expression of the three synaptic proteins (Figure 2C). Correspondingly, the protein levels of PSD95, SYP, and GAP43 were significantly elevated after exposure to lactate at both 2 and 4 mmol/L (Figure 2D). The aforementioned results suggested an upregulation of the synaptic proteins PSD95, SYP, and GAP43 by lactate in both hippocampal tissues and HT22 cells.

### 3.3. The Enhanced Expression of Synapse Proteins Induced by Lactate Is Associated with MCT2

**Figure 3:**
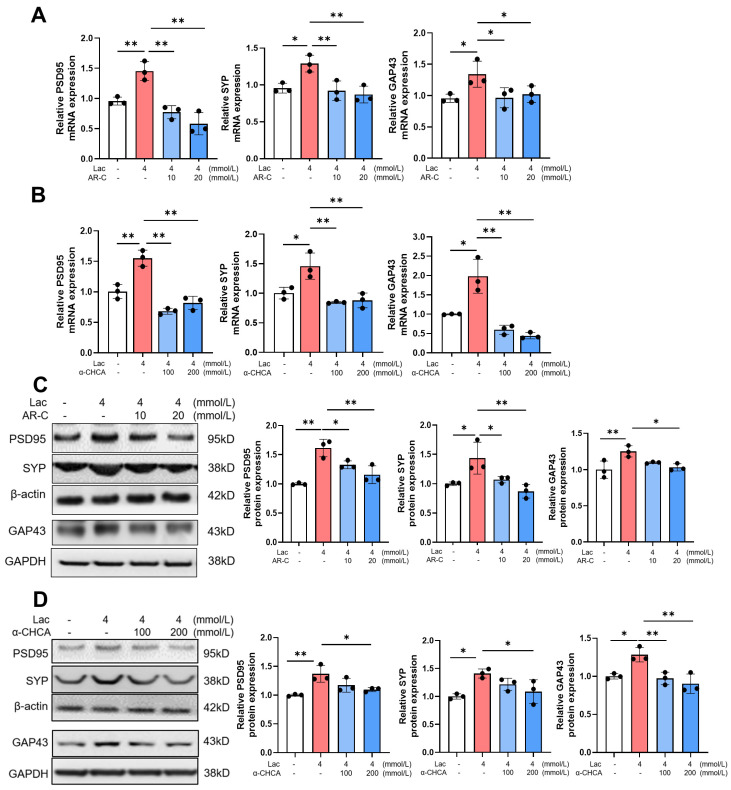
Lactate upregulates the levels of synaptic function-related proteins through MCT2. (**A**,**B**) qRT–PCR assays monitoring the expression of synaptic function-related proteins (PSD95, SYP, and GAP43) in lactate and MCT2 inhibitors AR-C (**A**) or α-CHCA (**B**) treated HT22 cells (*n* = 3, Ordinary one-way ANOVA, * *p* < 0.05; ** *p* < 0.01). (**C**,**D**) Western blot of the expression of synaptic function-related protein: PSD95, SYP, and GAP43 in lactate and MCT2 inhibitors AR-C158588 (**C**) or α-CHCA (**D**) treated HT22 cells (*n* = 3, Ordinary one-way ANOVA, * *p* < 0.05; ** *p* < 0.01).

MCT2 is the primary transporter expressed in neurons, facilitating the entry of lactate into cells for utilization [12,26]. To characterize the role of MCT2 in the regulation of synapse proteins by lactate, AR-C and α-CHCA were added to the cell culture medium along with lactate, followed by detection of the expression of PSD95, SYP, and GAP43. The results of qRT–PCR showed that, compared to the control cells, lactate enhanced the mRNA levels of PSD95, SYP, and GAP43, whereas MCT2 antagonists abolished this lactated-mediated effect (Figure 3A,B). Additionally, western blot assay also revealed that the increase in PSD95, SYP, and GAP43 proteins caused by lactate was inhibited by MCT2 antagonists (Figure 3C,D). These data indicate a facilitating role of MCT2 in the expression of synapse proteins induced by lactate.

### 3.4. Lactate Promotes Protein Lactylation

**Figure 4:**
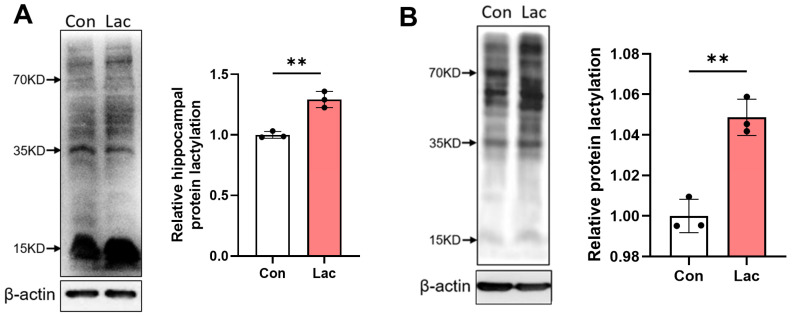
Lactate enhances protein lactylation. (**A**) Western blot of the expression of lactylation modified protein in the hippocampal samples from control and lactate-injected mice (*n* = 3, Student’s *t*-test, ** *p* < 0.01). (**B**) Western blot of the expression of lactylation modified protein in lactate-treated (4 mM) HT22 cells and control cells (*n* = 3, Student’s *t*-test, ** *p* < 0.01).

Recent studies have confirmed that lactylation, a post-translational modification, is a vital component of lactate function and is involved in neural excitation, inflammation, tumor proliferation, and other biological processes [17,21]. Lactate can induce the process of lactylation by adding a lactyl group to histone or some other protein’s lysine (K) residues [17]. To understand the mechanism of lactate in the regulation of spatial memory, we examined the changes in protein lactylation in lactate-treated hippocampal tissues and neurons using an anti-pan-lactyl-lysine (Kla) antibody. As shown in Figure 4A, lactate injection promoted the lactylation in total proteins extracted from the hippocampal tissues compared with the controls. In addition, an enhanced protein lactylation level was observed in the HT22 cells incubated with lactate (4 mM) versus the control cells (Figure 4B). Thus, an induction of protein lactylation by lactate was revealed.

### 3.5. MCT2 Is Involved in the Lactate-Mediated Lactylation

**Figure 5:**
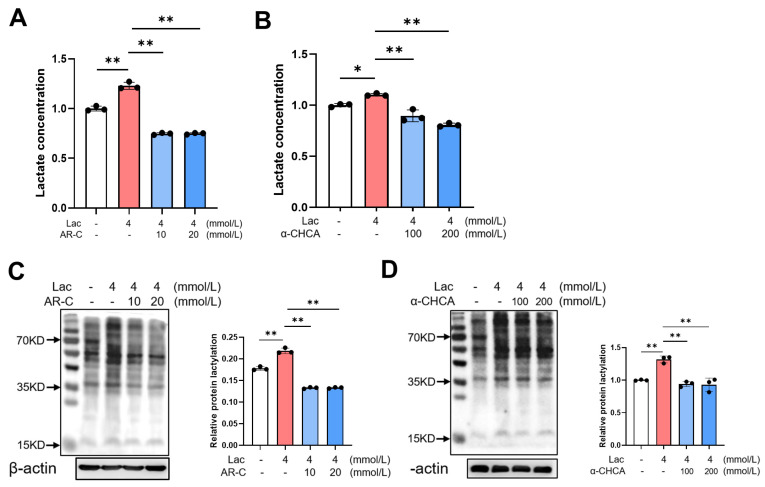
Increased levels of lactate-induced protein lactylation in neurons can be inhibited by MCT2 inhibitors. (**A**,**B**) Lactate levels in lactate and MCT2 inhibitors AR-C158588 (**A**) or α-CHCA (**B**) treated HT22 cells (*n* = 3, Ordinary one-way ANOVA, * *p <* 0.05; ** *p <* 0.01). (**C**,**D**) Western blot of the expression of lactylation modified protein in lactate and MCT2 inhibitors AR-C158588 (**C**) or α-CHCA (**D**) treated HT22 cells (*n* = 3, Ordinary one-way ANOVA, ** *p <* 0.01).

We further investigated the involvement of MCT2 in the protein lactylation induced by lactate by using AR-C and α-CHCA as inhibitors. The levels of intracellular lactate and lactylation were examined. Compared to the HT22 cells treated with lactate, the levels of intracellular lactate decreased after AR-C or α-CHCA treatment, suggesting an inhibition of lactate transport into cells (Figure 5A,B). Furthermore, both AR-C and α-CHCA suppressed the protein lactylation induced by lactate (Figure 5C,D). Taken together, the data indicate that inhibiting the function of MCT2 impedes the transport of lactate into neurons and thus limits the protein lactylation induced by lactate.

### 3.6. RNA-Seq Analysis Identifies Gene Expression Profile in the Hippocampus Regulated by Lactate

**Figure 6:**
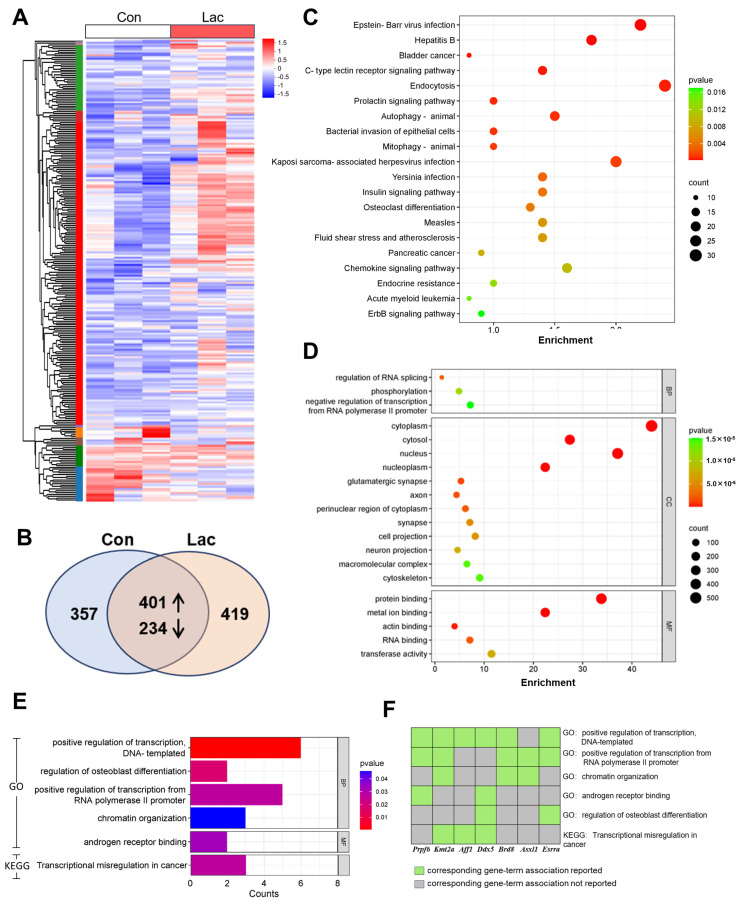
Summary of RNA-seq data from lactated-injected hippocampal tissues and controls. (**A**) Heat map analysis of gene expression changes in hippocampal tissues injected with lactate (*n* = 3). (**B**) Total number of significantly differentially expressed genes and the percentage of up- and down-regulated genes. (**C**) Results of KEGG analysis of differentially expressed genes (Top 20 categories). (**D**) Results of GO analysis of differentially expressed genes (Top 20 categories). (**E**) GO and KEGG analyses of the top-20 DEGs. (**F**) The genes included in GO and KEGG categories.

RNA-seq was performed to identify the DEGs between the hippocampal tissues injected with lactate and the controls. Hierarchical clustering analysis revealed a total of 1411 DEGs (change > 2-fold, *p* < 0.05) in the lactated-infused hippocampus, of which 419 genes were uniquely induced and 357 genes were fully inhibited by lactate. Besides, 401 genes were enriched, while 234 genes were decreased in the lactate group compared to the control group (Figure 6A,B), suggesting that lactate is more likely to stimulate gene expression. Aiming to predict the functions of the DEGs, KEGG analysis and GO analysis were applied. The results of KEGG analysis indicated potential involvement of these DEGs in the pathways correlated with the immune and endocrine systems, such as the C-type lectin receptor signaling pathway, Prolactin signaling pathway and Insulin signaling pathway (Figure 6C). GO analysis revealed lactate’s regulation of genes in both cytoplasm and nucleus, with a particular emphasis on the glutamatergic synapse and axon. The molecular functions most strongly associated with the DEGs were related to binding activities, and the biological processes regulated by the DEGs involved “regulation of RNA splicing”, “phosphorylation”, and “transcription” (Figure 6D). Furthermore, GO and KEGG analyses of the top-20 DEGs were conducted, aiming to unravel further insights into the molecular mechanism underlying the impact of lactate on neuronal and memory functions. The results indicated a significant association between these genes and the transcriptional process (Figure 6E and Supplementary Table S3). Notably, DEGs including Kmt2a, Prpf6, Aff1, Ddx5, Brd8, Asxl1, and Esrra have been previously reported as involved in this biological process (Figure 6F).

## 4. Discussion

In previous studies, lactate has been considered as metabolic by-product, and most studies have focused on the side effects of lactate on organisms. However, as a metabolic small molecule, lactate has a comprehensive effect on organisms, and positive mechanisms are increasingly being investigated [8,27,28].

A growing amount of research suggests the significance of lactate in maintaining cognitive function [10,29,30]. Recent studies have demonstrated that lactate produced by astrocytes can serve as the primary energy source for active neurons [12,31], which may prefer lactate to glucose under equal conditions [32]. In an Alzheimer’s disease model of APP/PS1 mice, decreased levels of brain parenchymal lactate and various MCT subtypes were observed [33]. Furthermore, lactate exhibits neuro-protective properties in pathological conditions, including hypoglycemia, ischemic attack, and traumatic brain injury [34,35]. It was found that lactate induces hippocampal neurogenesis [30], which is mainly concentrated in the sub-granular zone (SGZ) of the dentate gyrus of the hippocampus. Lactate induces NPC proliferation in the SGZ by stimulating the ERK1/2 or PI3K/Akt pathway, which is related to the proliferation of neurons regulated [36]. The hippocampal CA1 region is responsible for the generation and maintenance of long term memory and plays an important role in spatial memory and behavioral control. Lactate may play a protective role against traumatic brain injury by promoting the expression of MCT2 and plasticity-related proteins in the hippocampal CA1/CA2 region [37]. To further investigate the role of lactate under normal physiological conditions, we elevated hippocampal lactate levels via cannula needle injection and observed improved spatial memory ability in mice. Therefore, supplementing lactate levels under non-pathological conditions may enhance cognitive performance. In a future study, we will further investigate the functional characteristics of lactate in different hippocampal regions.

Lactate transport occurs via MCT, which are proton-linked membrane carriers that transport monocarboxylates through cell membranes [38,39]. In the central nervous system (CNS), MCT2 is predominantly expressed on the surface of neurons and exhibits a preference for lactate uptake and utilization [26]. In a study conducted by Suzuki et al., it was observed that knockdown of MCT2, the neuronal lactate transporter, in the hippocampus of mice resulted in long-term memory deficits [12]. Notably, lactate supplementation failed to reverse these memory deficits, indicating the crucial role of neuronal lactate uptake in long-term memory formation. Being transported into neurons in a MCT2-dependent manner, lactate may affect synaptic plasticity by affecting the expression of Arc, Egr1 and BDNF related genes, or by affecting ATP production to regulate neural excitability [10,23,40,41,42]. In the central nervous system, MCT2 expression was detected in the cell body and axon surface of almost all MAP2-positive cells [38]. Interestingly, MCT2 was found to be expressed at the synapses of glutamatergic neurons and colocalized with post-synaptic glutamate receptors in the cerebral cortex, hippocampus and cerebellum, but not at the synapses of GABAergic neurons [43,44]. Our study revealed that lactate administration increased the expression of synaptic function-related proteins PSD95, SYP, and GAP43, which are vital for synaptic transmission and significantly contribute to memory formation and retention. More intriguingly, inhibition of MCT2 resulted in decreased neuronal uptake of lactate and attenuated the lactate-induced expression of PSD95, SYP, and GAP43. Our findings indicate that lactate exerts its cognitive-enhancing effects through MCT2.

Recent studies have revealed that lactate plays a crucial role in cellular regulation by contributing to epigenetic regulation through covalent modification of protein lysine residues [14,17]. Lactylation, a novel post-translational modification first reported in 2019, involves the modification of histone lysine residues [17,45]. Recent high-throughput omics and experimental studies have demonstrated that lactylation can also occur on non-histone proteins [46] and has been implicated in various biological processes, including neural excitation [22], inflammation [17,19], and tumorigenesis [20]. A study conducted on mice found that neural excitation and behavior-related stimuli increased lactate levels and resulted in the lactylation of 63 proteins in the brain, offering evidence of widespread protein lactylation throughout the brain and its regulation by lactate production [22]. In this study, we investigated the impact of lactate on protein lactylation in the mouse hippocampus using a highly specific lactic-modified pan-antibody to label lactylated proteins. Our findings reveal that lactate administration significantly increased protein lactylation around the position of 13 KD–17 KD, corresponding to the molecular weight of histone proteins. Immunoblotting analysis also showed increased protein bands at different sizes following lactate injection, indicating non-histone lactylation changes. However, further investigations are necessary to identify the specific targets of lactylation.

Numerous studies have demonstrated the close relationship between protein lactylation and gene expression regulation [18,47]. Histone lactylation can directly interact with gene promoter regions, thereby influencing gene transcription. Several types of histone lactylation, such as H3K18 [48], H4K12 [49], and H3K9 lactylation [50], have been identified as promoting gene expression. In this experiment, we found that lactate can promote the degree of protein lactate modification, and the degree of histone lactate changes significantly. The expression of synaptic function-related proteins, such as PSD95, is affected by epigenetic regulation [51,52], and histone lactylation is one of the important types of epigenetic modification. Therefore, we speculate that lactate may regulate the expression of synaptic function-related proteins, such as PSD95, through epigenetic modification by regulating the level of histone lactylation. Furthermore, nonhistone protein lactylation has also been found to regulate gene expression. For example, high levels of lactate induce lactylation of Yin Yang-1 (YY1), a transcription factor, at lysine 183 (K183), leading to enhanced FGF2 transcription and angiogenesis promotion [53]. In this study, we employed RNA-seq technology to investigate the impact of lactate on hippocampal gene expression. The data revealed that a significant number of genes exhibited increased expression following lactate injection (820 out of 1411 DEGs), consistent with the promotion of gene expression through lactate-induced lactylation. Besides, bioinformatics analysis of the top-20 DEGs indicated their strongly association with the biological processes of “transcription”. Among the top-20 DEGs, Kmt2a showed an involvement in the most GO terms. Kmt2a (MLL1) is a H3K4 methyltransferase. Research has demonstrated that conditional knockout of the hippocampal Kmt2a gene in mice can lead to significant impairment in spatial memory, and this process may associate with its regulation of synaptic plasticity genes expression [54]. Therefore, the role of Kmt2a in lactate-induced cognitive enhancement and the impact of lactylation on it deserve further investigation.

This study found that lactate was involved in the promotion of spatial memory ability by mediating the changes in the level of protein lactate modification and the extensive changes in gene expression. However, there are also some limitations in our study. We have not found a direct molecular target for lactate to promote spatial memory ability, and we have not explored the molecular biological mechanism of how lactate is involved in the regulation of neuronal synaptic function. In the following experimental work, we will continue to try to explore the possible biological mechanism of lactate and conduct exploration and verification, so as to provide new ideas for exploring the regulatory effect of lactate on cognitive function. In the experiment, the number of animals in some experimental groups is small, so the number of experiments can be expanded in the following experiments to make the experimental results more solid and credible.

## 5. Conclusions

**Figure 7:**
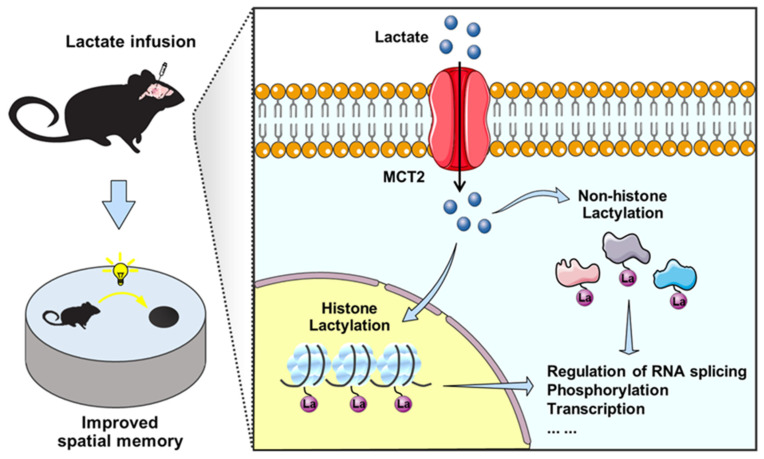
A schematic model of the role of lactate in regulating spatial memory in mice.

In summary, this study found that elevated levels of lactate in the hippocampus enhance spatial memory and key synaptic protein expression in mice. Lactate administration induces extensive protein lactylation and gene expression changes, providing insight into the mechanism underlying lactate-mediated spatial memory improvement (Figure 7). These findings highlight the beneficial effects of lactate on memory regulation and provide new insights for the treatment of cognitive impairment.

## Data Availability

The datasets generated during and/or analysed during the current study are available from the corresponding author on reasonable request. The data are not publicly available due to specific ethical and privacy considerations.

## Supplementary Materials

The following supporting information can be downloaded at: https://www.mdpi.com/article/10.3390/brainsci14040327/s1, Table S1. Primer List; Table S2. Antibody List; Table S3. Top-20 DEGs in the hippocampus infused with lactate compared to the controls.
