# Supplementary material for: Hippocampal Lactate-Infusion Enhances Spatial Memory Correlated with Monocarboxylate Transporter 2 and Lactylation
Source: Brain Sci. 2024 Mar 28;14(4):327. doi: 10.3390/brainsci14040327 (PMC11048250; doi:10.3390/brainsci14040327)
Supplement: Supplementary file 1 [file brainsci-14-00327-s001.zip › brainsci-2936324-supplementary.pdf]

Hippocampal lactate-infusion enhances spatial memory correlated with MCT2 and lactylation

Yuhan Wu[1], Hui Hu[1], Weiwei Liu[1, 2], Yun Zhao[1], Fang Xie[1], Zhaowei Sun[1], Ling Zhang[1], Huafeng Dong[1], Xue Wang[1,*], Lingjia Qian[1,*]

[1] Beijing Institute of Basic Medical Sciences, Academy of Military Medical Sciences, Beijing, 100039, China

[2] North China University of Science and Technology, TangShan, 063210, Hebei, China

*** Correspondence:**

Lingjia Qian, MD, PhD and Xue Wang, PhD

newjia@vip.sina.com (Lingjia Qian), snowwang0326@foxmail.com (Xue Wang)

Supplementary Tables

Supplementary Table 1. Primer List.

Gene	Primer sequence	
MCT2	Forward primer (5’-3’)	TGGGCCAAGTCCAAATACTAAGA
	Reverse primer (5’-3’)	TGCTGTTGATACCTTACTGGCT
GAP43	Forward primer (5’-3’)	AGATGGTGTCAAGCCGGAAG
	Reverse primer (5’-3’)	CGCCTTTGAGCTTTTTCCTTGT
PSD95	Forward primer (5’-3’)	TACCAAGATGAAGACACGC
	Reverse primer (5’-3’)	CTGCAACTCATATCCTGGG
SYP	Forward primer (5’-3’)	GACGTTGGTAGTGCCTGTGA
	Reverse primer (5’-3’)	GCACAGGAAAGTAGGGGGTC
β-actin	Forward primer (5’-3’)	CGATGCCCTGAGGCTCTTTT
	Reverse primer (5’-3’)	GAGGTCTTTACGGATGTCAACG

Supplementary Table 2. Antibody List.

Antigens	Manufacturer	Application
PSD95	Immunoway, USA	1:1000 for WB
SYP	Immunoway, USA	1:1000 for WB
GAP43	Immunoway, USA	1:1000 for WB
MCT2	Immunoway, USA	1:1000 for WB
L-Lactyl Lysine	PTM BIO, China	1:1000 for WB
β-actin	Abclonal Technology, China	1:5000 for WB
GAPDH	Proteintech, China	1:5000 for WB

WB, western blotting

Supplementary Table 3. Top-20 DEGs in the hippocampus infused with lactate compared to the controls.

Gene_id	Gene name	Log2FC	p value	Regulate
ENSMUSG00000028936	Rpl22	11.7760	0.0026	up
ENSMUSG00000002455	Prpf6	10.8907	0.0010	up
ENSMUSG00000002028	Kmt2a	10.8117	0.0057	up
ENSMUSG00000025512	Chid1	10.2717	0.0086	up
ENSMUSG00000030737	Slco2b1	10.2145	0.0000	up
ENSMUSG00000061578	Ksr2	10.1764	0.0000	up
ENSMUSG00000029313	Aff1	10.1553	0.0093	up
ENSMUSG00000026792	Lrsam1	9.7840	0.0123	up
ENSMUSG00000020719	Ddx5	9.6641	0.0000	up
ENSMUSG00000024012	Mtch1	9.4986	0.0000	up
ENSMUSG00000030223	Ptpro	9.3354	0.0000	up
ENSMUSG00000003778	Brd8	9.3150	0.0000	up
ENSMUSG00000042548	Asxl1	9.2926	0.0174	up
ENSMUSG00000020149	Rab1a	-10.1279	0.0000	down
ENSMUSG00000068037	Mas1	-10.0971	0.0000	down
ENSMUSG00000024083	Pja2	-9.9027	0.0000	down
ENSMUSG00000024955	Esrra	-9.6327	0.0137	down
ENSMUSG00000055003	Lrtm2	-9.4800	0.0000	down
ENSMUSG00000020886	Dlg4	-9.3499	0.0001	down
ENSMUSG00000031691	Tnpo2	-9.3290	0.0170	down
